# Identification and expression of *Hop*, an atypical homeobox gene expressed late in lens fiber cell terminal differentiation

**Published:** 2007-01-26

**Authors:** Oleg Vasiliev, Simon J. Rhodes, David C. Beebe

**Affiliations:** 1 Department of Ophthalmology and Visual Sciences, Washington University, St. Louis, MO; 2 Department of Cell Biology and Physiology, Washington University, St. Louis, MO; 3 Shemyakin and Ovchinnikov Institute of Bioorganic Chemistry, Russian Academy of Sciences, ul. Miklukho-Maklaja 16/10, V-437 Moscow, Russia; 4 Department of Cellular and Integrative Physiology, Indiana University School of Medicine, Indiana University-Purdue University Indianapolis, IN

## Abstract

**Purpose:**

To identify transcripts expressed late in lens fiber cell maturation that might regulate fiber cell fusion, organelle degradation, or other events associated with the maturation of lens fiber cells.

**Methods:**

cDNA libraries were prepared from microdissected regions of chicken embryo lenses using a PCR-based method. Subtractive hybridization was used to identify transcripts expressed exclusively in fiber cells that had detached from the lens capsule. Database searches and PCR amplification with degenerate primers were used to identify human, mouse, rat, rabbit, and bovine orthologs of one such sequence and to confirm its expression in the lenses of these animals. The ability of in vitro-transcribed and translated protein to bind DNA was assessed by mobility shift assays. The locus encoding this transcript and an area about 6 kb upstream of the translation start site were sequenced. The microscopic morphology of lenses from mice in which the locus encoding this protein had been disrupted by the insertion of a nuclear-targeted bacterial lacZ sequence were analyzed. Gene expression was analyzed by PCR, in situ hybridization, and by staining for β-galactosidase activity in lenses expressing *lacZ* in place of the coding sequence. Knockout lenses expressing green fluorescent protein in a mosaic pattern were sectioned in the equatorial plane and viewed with a confocal microscope to assess the presence of cell-cell fusions during fiber cell maturation.

**Results:**

Subtractive hybridization identified transcripts encoding Hop, a short, atypical homeodomain-containing protein that had previously been shown to be an important regulator of gene expression in the heart and lung. Chicken Hop did not bind to known homeodomain-binding sequences in DNA. In chicken embryos, *Hop* transcripts were first detected at E6. At all stages analyzed, *Hop* mRNA was only detected in cells that had detached from the lens capsule. Mice in which the *Hop* coding sequence was replaced with nuclear-targeted β-galactosidase showed that Hop was expressed in the mouse lens in a similar pattern to the chicken lens. Characterization of lenses from mice lacking *Hop* revealed no morphological phenotype and no apparent defects in the degradation of nuclei or fiber cell fusion during fiber cell maturation.

**Conclusions:**

The expression pattern of *Hop* provides the first evidence that new transcription is initiated in lens fiber cells after they detach from the capsule. *Hop* may be the first of a class of genes with this pattern of expression. Although lens abnormalities have yet to be identified in mice lacking *Hop*, the genomic sequences that regulate Hop expression in the lens may be useful for expressing exogenous transcripts selectively in fiber cells just before they fuse with their neighbors and degrade their organelles.

## Introduction

**Figure 1:**
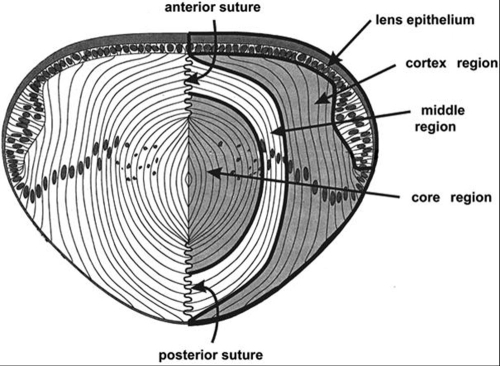
Diagram of lens regions. Diagram representing a section through the center of a lens showing the regions of the fiber mass that were dissected to produce region-specific cDNA libraries. Fiber cells in the cortex region are still in the process of elongation and are attached at their basal ends to the lens capsule, the lens basement membrane. Cells in the middle region have completed the process of elongation and have detached from the capsule. The apical and basal ends of these cells abut the ends of fiber cells from the other side of the lens at the anterior and posterior sutures. Cells in the core region have degraded their nuclei and other membrane-bound organelles.

The lens is composed of two types of epithelial cells: A sheet of cuboidal cells, the lens epithelium, covers its anterior surface, and post-mitotic, elongated fiber cells comprise the bulk of the lens (Figure 1). Stimulation by factors present in the vitreous body causes epithelial cells near the lens equator to withdraw from the cell cycle and differentiate into lens fiber cells. Differentiating fiber cells elongate and initiate the transcription of genes that encode a distinct array of abundant membrane, cytoskeletal, and cytoplasmic proteins. The accumulation of high concentrations of cytoplasmic proteins (crystallins) in fiber cells is important for the transparency and refractive power of the lens. Some crystallins, cytoskeletal, and membrane proteins are found primarily in lens cells, or are present only at very low levels in non-lens tissues [1-7].

Lens fiber cells undergo remarkable morphological changes during their differentiation. Fiber cells first elongate to many times their original length, extending to over 140 μm per day in the chicken embryo [8]. As they elongate, the anterior and posterior ends of the fiber cells extend beneath the lens epithelium and along the posterior lens capsule toward the optical axis. When the ends of these cells approach the anterior and posterior poles of the lens, they meet elongating fiber cells extending from the other side, resulting in the formation of the anterior and posterior sutures (Figure 1). Once the cells stop elongating, they become buried beneath the next group of elongating fiber cells. Soon after the fiber cells detach from the posterior capsule, the composition of their cell-cell adhesion proteins changes [9], their lateral membranes become interdigitated [8] and partially fuse with the membranes of neighboring fiber cells [8,10], and all intracellular, membrane-bound organelles are degraded [11-17]. Mature fiber cells persist in this state for the life of the organism.

Many of the genes that are preferentially expressed in lens fiber cells have been identified, cloned, and sequenced, and their promoters used to express foreign genes in the lenses of transgenic animals [18,19]. The products of all these "fiber-specific" genes are detected at or soon after the initiation of fiber cell differentiation. To date, only a few transcripts have been identified that are preferentially expressed late in fiber cell differentiation [9,20], and no mRNAs have been identified that are expressed only at this stage.

To identify molecules that might regulate or be required for the final stages of fiber cell differentiation, we used subtractive hybridization between cDNA libraries created from chicken embryo lens fiber cells before and after they detached from the lens capsule. The open reading frame of one of the transcripts that was selectively expressed after fiber cells detached from the capsule consisted of 73 amino acids, 60 of which had strong sequence similarity to the homeodomain consensus. This gene was previously named *Hop*, for "homeodomain-only protein" [21,22].

## Methods

### Animals and surgical procedures

Animals were treated in accordance with the guidelines of the U.S. Public Health Service under a protocol approved by the Washington University Animal Studies Committee. Mice were maintained in an animal facility accredited by the Association for Assessment and Accreditation of Laboratory Animal Care (AAALAC). Fertile chicken eggs were obtained from CBT Farms (Chestertown, MD) and incubated in a humidified, forced-draft incubator at 38 °C. Embryos at different stages of development were removed, their lensers were fixed, and sectioned at 500 μm with a tissue slicer (OTS-4000; Electron Microscopy Sciences, Warrington, PA), and examined for the distribution of *Hop* mRNA by in situ hybridization. Mice in which the *Hop* coding sequence was replaced with DNA encoding a nuclear-targeted form of *E. coli* β-galactosidase [22] were genotyped by PCR. Whole lenses were stained for β-galactosidase activity according to directions described in reference [23], embedded in glycol methacrylate, and sectioned at 1 μm. *Hop* knockout mice were mated with TgN(GFPU)5Nagy strain of mice, which express green fluorescent protein (GFP) in a mosaic pattern, to determine whether lens cells lacking Hop fused with their neighbors during fiber cell maturation [24]. Lenses lacking Hop were fixed in 10% formalin and sectioned perpendicular to the long axis of the fiber cells. GFP fluorescence was viewed with a Zeiss LSM 510 confocal microscope.

### Overview of the subtractive hybridization method

The procedures used were modified from previous reports [25,26]. RNA was prepared from microdissected regions of the lens fiber cells and region-specific cDNA pools were synthesized. The cDNAs were amplified by PCR, made single-stranded (tester), and annealed with excess biotinylated, single-stranded cDNA prepared from a different region of the lens (driver). The biotinylated complex was removed with magnetic streptavidin beads and the remaining cDNA was cloned into a bacterial plasmid. Bacterial clones were screened with labeled probes prepared from both regions of the lens to confirm the effectiveness of the subtraction.

### Preparation of region-specific libraries

In chicken embryos, organelle degradation in the central fiber cells begins at E12 [11]. After this age, lenses contain elongating fiber cells, fiber cells that have stopped elongating and detached from the capsule but not yet degraded their organelles, and mature fiber cells with no organelles (Figure 1). To isolate mRNA from these populations, we removed E15-16 lenses from the eye, embedded them in 4% agar, and cut 500 μm slices parallel to the optic axis with a tissue slicer. Slices that included the center of the lens were dissected into three regions: "cortex," which contained elongating fiber cells, "middle," which contained fiber cells that had detached from the capsule yet still contained organelles, and "core," which contained the fiber cells that had already lost their organelles (Figure 1). The following primer sequences for PCR reactions were used: T primer: 5'-GTG CCT CTA GAT TTT TTT TTT-3'; TC primer: 5'-GTG CCT CTA GAT TTT TTT TTT GGA TCC CCC CCC CC-3'; C primer: 5'-TTT TCA CGG ATC CCC CCC CCC-3'; X primer: 5'-GTC CGG CCA ACG GTA TGG TG-3'; XT primer: 5'-GTC CGG CCA ACG GTA TGG TGC CTC TAG ATT TTT TTT TT-3'; and XC primer: 5'-GTC CGG CCA ACG GTA TGG TGC ACG GAT CCC CCC CCC C-3'.

Total RNA from these regions was extracted using the standard guanidine thiocyanate procedure as described in reference [27]. Total RNA (0.25-1 μg) was heat-denatured at 65 °C for 5 min and annealed at room temperature for 5 min with 5 pM of T-primer in a total volume of 20 μl containing 1X RT buffer (50 mM Tris-HCl, pH 8.3, 6 mM MgCl_2_, 75 mM KCl, 1 mM dithiothreitol, 1 mM of each dATP, dTTP, dCTP, and dGTP), and 1 U RNase inhibitor (Promega, Madison, WI). After addition of 200 units of Moloney murine leukemia virus reverse transcriptase (SuperScript II; Life Technologies, Gaithersburg, MD), incubation was continued at 42 °C for 1 h, followed by 94 °C for 5 min, using a programmable thermal cycler (PTC-100TM; MJ Research, Watertown, MA). The tube was spun briefly and the cDNA was purified from the T-primer and dNTPs by ultrafiltration through a Microcon-100 concentrator (Millipore, Billerica, MA). Purified cDNA (5 μl aliquots) was oligo-dG-tailed at 37 °C for 0.5-1.5 h in a total reaction volume of 15 μl containing 1X terminal deoxynucleotidyl transferase (TdT) buffer (50 mM sodium cacodylate, pH 7.2, 0.1 mM 2-mercaptoethanol, 1 mM CoCl_2_), 400 mM dGTP and 2 U TdT. Higher TdT concentration (5-20 U) as well as longer time of incubation can significantly reduce the amount of DNA available for subsequent amplification with TC- and T-primers. For this reason, 4 μl aliquots of DNA were removed every 30 min of incubation and submitted to 18 cycles of "hot-start" PCR in 50 μl of 1X PCR buffer (40 mM Tricine-KOH, pH 9.2, at 20 °C; 10 mM potassium acetate, 3 mM MgCl_2_; 50 mg/ml BSA, 200 mM of each dATP, dTTP, dCTP, and dGTP), 5 pM TC primer, 20 pM T primer and 1.5 U Taq Polymerase. The first cycle of PCR was 94 °C for 30 s, 52 °C for 1 min, 72 °C for 1.5 min, followed by 16 cycles (94 °C for 10 s, 56 °C for 20 s, and 71 °C for 1.5 min). The final cycle had 5 steps: 94 °C for 10 s, 56 °C for 20 s, 71 °C for 2 min, 56 °C for 20 s, and 71 °C for 2 min. The five-step PCR was used during the last cycle for converting single-stranded "pan-like" DNA to double-stranded products. Full-length TC-T cDNA was prepared using the same conditions and cycle parameters except the elongation time was extended to 5 min and 0.05 U of Pfu polymerase was added to the PCR mix [28].

### Preparation of biotinylated T-C cDNA

To obtain biotinylated "middle" and "cortex" T-C cDNA, 10 ng of the original TC-T cDNA was amplified through 10 cycles of PCR with 5'-end biotinylated T- and C-primers (Integrated DNA Technologies), the product purified from the primers and unincorporated nucleotides with a PCR purification kit (Promega), ethanol precipitated and resuspended in 12 μl of deionized water.

### Driver preparation

About 7 μg of sense and antisense cDNA (driver) were prepared from 5 μl (2 μg) of biotinylated T-C cDNA (Integrated DNA Technologies) by five cycles of asymmetric amplification with 100 pM of biotinylated C-primer (for sense) or biotinylated T-primer (for antisense) in five tubes with a total volume 250 μl. The PCR reaction was stopped by the addition of 2 μl of 0.5 M EDTA, pH 8.0, and frozen at -20 °C.

### Tester (tracer) preparation

Sense and antisense tracer cDNA were prepared from 1 μl of biotinylated T-C cDNA by additional 5 cycles of asymmetric PCR with 20 pM of un-biotinylated XC-primer (for sense) or XT-primer (for antisense) in 50 ml of PCR buffer. The PCR reaction was stopped by addition 1 μl of 0.5 M EDTA pH 8.0 and frozen at -20 °C.

### Subtractive hybridization

For each subtraction, two samples were prepared: one containing 5 μg of sense driver and 250 ng of antisense tracer and the second containing antisense driver and sense tracer. After undergoing phenol-chloroform extraction and precipitation with ethanol, each cDNA sample was dissolved in 400 μl of deionized water and purified from the primers by 5X filtration with Microcon-100 filters. Purified cDNA was precipitated with ethanol and resuspended in 4 μl of hybridization buffer (50 mM HEPES, pH 8.3; 0.5 M NaCl; 0.05 mM EDTA, pH 8.0), overlaid with mineral oil, heated 2 min at 95 °C and incubated overnight at 68 °C. The hybridization mix was diluted in 400 μl of NTE buffer (10 mM Tris-HCl, pH 8.0, 0.5 M NaCl, and 1 mM EDTA) and the aqueous phase was transferred to a fresh tube with 100 μl of streptavidin-beads (Dynal Biotech, Lake Success, NY) in NTE buffer (the beads were washed 3X in NTE buffer before use). After 5 min incubation at room temperature, the beads and bound DNA were removed with a magnet, and the remaining cDNAs were subjected to a second round of purification with streptavidin beads. After purification, the two samples were combined, mixed with 1 μg of each sense and antisense driver, precipitated with ethanol, dissolved in 4 μl of hybridization buffer and used for second step of hybridization at 68 °C, overnight. The second hybridization mix was purified twice with streptavidin beads. PCR was performed with 2 μl of the remaining cDNA in 50 μl of PCR buffer containing 10 pM of X-primer using the following parameters: 72 °C for 3 min, then 25-30 cycles of 94 °C for 12 s; 56 °C for 20 s; 72 °C for 2 min. The PCR reaction mixture was diluted 500 times and subjected to additional 15-17 rounds of PCR with T- and C-primers. Purified product of this secondary PCR was digested with *Xba* I and *Bam* HI endonuclease (Roche Applied Science, Indianapolis, IN) and inserted into a pcDNA3.1(-) vector (Invitrogen, Carlsbad, CA). For differential screening, 96 individual clones from the subtracted "middle" library were replicated and hybridized with DIG-labeled probes synthesized by PCR from "cortex" and "middle" subtracted cDNA. Plasmids from clones that reacted only or preferentially with the "middle" library were sequenced.

### Genomic sequencing

Sequencing of the region upstream of the *Hop* translation start site was accomplished by genomic walking. Chicken genomic DNA was extracted and digested with one of several restriction enzymes that generate 5' overhangs. The genomic fragments were ligated to double-stranded anchor primers with the appropriate 3' overhangs using the Rapid DNA Ligation Kit (Roche Applied Science), and PCR products were amplified with primers designed against the *Hop* coding sequence and the sequence of the anchor primer. The longest PCR fragments were cloned and sequenced using standard methods. Potential transcription factor binding sites in the genomic sequence upstream of the translation start site were identified with P-Match, a public version of Match (Biologische Datenbanken GmbH, Wolfenbüttel, Germany), using the stringency cutoff selection to minimize the identification of false positive matches.

### Tests of Hop DNA binding

Electrophoretic mobility shift assays (EMSA) were performed as described [29]. Radiolabeled Hop protein was synthesized in vitro using TNT Quick Coupled rabbit reticulocyte lysate reagents (Promega, Madison, WI) and ^35^S-methionine (Amersham Pharmacia, Piscataway, NJ). Substrate DNA was 0.5 mg of a plasmid containing *Hop* cDNA. ^32^P-labeled oligonucleotides representing binding sites for transcription factors were as follows: LHX3 LIM-class homeodomain site, 5'-GAT CCC AGA AAA TTA ATT AAT TGT AA-3' (LBC) [29]; paired-class homeodomain site, 5'-TCC GAC TAA TTG AAT TAG CGA GA-3' (PRD) [30]; bicoid-class homeodomain site, 5'-GAT CCG CAC GGC CCA TCT AAT CCC GTG GGA TC-3' (BIC) [31]; Pit-1 POU-class homeodomain site, 5'-GAT CCT ATG TGC TCA AAG TTC AGG TAT GAA TAT AAA GGA TC-3' (PIT) [32]; and a MyoD basic helix-loop-helix site, 5'-GGG AAA GGA TCT GAC AGG TGG CCC CAG CCC TCG G-3' (MD).

### Amplification of *Hop* sequences using degenerate PCR primers

cDNA prepared from the lenses of several species was amplified with degenerate primers based on the sequence of chicken *Hop*. The primers were: 5'-GAT TCC ACC ACG CTG TGY CTN ATY GC-3' and 5'-CCA CTT BGC CAG NCG YTG YTT-3' where Y is C or T, N is A,G,T or C and B is C, G, or T. PCR products were cloned and sequenced by standard methods.

### Northern blotting

Total RNA from E15 lens fiber masses was separated by agarose gel electrophoresis, transferred to nylon membranes (Roche Applied Science, Indianapolis, IN), and probed with digoxigenin-labeled antisense riboprobes derived from the chicken *Hop* sequence by following directions given in the manual provided with the riboprobe kit. Bands were visualized with a peroxidase-labeled antibody to digoxigenin and chemiluminescent detection (Roche Applied Science).

In situ hybridization was performed using standard techniques for whole mount staining [33] on whole lenses (E6-E8) or about 500 μm-thick sections of formaldehyde fixed lenses (>E8). Lenses were fixed for about 1 h, washed in PBS, and stained whole or sectioned using an OTS-4000 tissue slicer. Sections were stained with antisense or sense digoxigenin-labeled riboprobes derived from the full length chicken *Hop* cDNA sequence. An alkaline phosphatase-conjugated antibody to digoxigenin and 5-bromo-4-chloro-3-indolyl phosphate/Nitro blue tetrazolium were used for color development (Roche Applied Science).

## Results

To identify genes expressed late in fiber cell maturation we used a PCR-based method to prepare cDNA libraries from microdissected regions of E15-16 chicken lenses (Figure 1) and performed subtractive hybridization to identify cDNAs that are selectively expressed in fiber cells that had detached from the lens capsule, yet still contained organelles. Several clones were identified that were enriched or were expressed exclusively in mature fiber cells. One of these cDNAs encoded vinculin (GenBank NM_205441), a transcript that we had previously found to increase after fiber cells detach from the lens capsule [9]. Most other clones from this library encoded genes that were differentially, but not exclusively, expressed in mature fiber cells. One clone encoded a sequence that was expressed selectively in detached fiber cells but at low levels. A few ESTs for this transcript have been identified, the longest being GenBank accession number CN228064. Since this transcript was expressed at a low level in the lens, it was not examined further. Another transcript was expressed at high levels only in fiber cells that had detached from the capsule. It contained a short open reading frame encoding 73 amino acids with sequence similarity to the homeodomain transcription factors (GenBank NM_204556). The mouse ortholog of this gene (*Hop*) was recently shown to be expressed in heart development and to modulate the activity of other transcription factors [21,22,34].

Sequence analysis and database searches revealed that chicken *Hop* differs at several locations from the homeodomain consensus and is not sufficiently similar to any of the known homeodomain sequences to be grouped in one of the homeodomain "superclasses" [35]. The greatest similarity of *Hop* to a characterized homeodomain is 47% amino acid identity with the Pitx homeoprotein of the cephalochordate *Branchiostoma belcheri* [36], although it is nearly as closely related to many other homeodomains of the "paired" superclass. The Hop homeodomain is 61 amino acids, containing a valine between the first and second helix, a characteristic sometimes present in diverged homeodomains [35].

**Figure 2:**
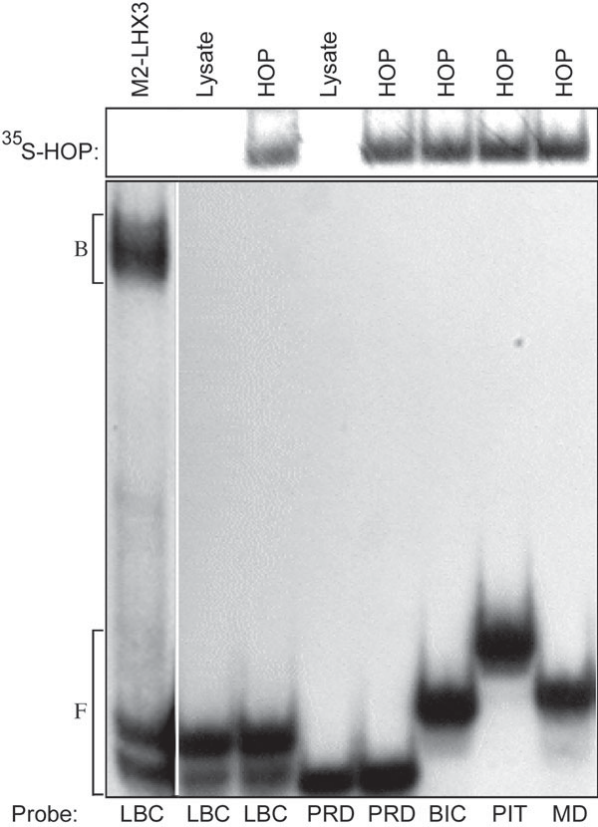
Hop does not bind to homeobox sequences. Electrophoretic mobility shift assay using radiolabeled oligonucleotide probes representing transcription factor binding sites. Probes were incubated with the indicated ^35^S-labeled in vitro translated proteins, and the bound complexes (B) were separated from the free probe (F) by electrophoresis. Unprogrammed lysate was used as a negative control (lysate). Bacterially expressed M2-LHX3 was used as a positive control [37]. Abbreviations: LBC=LHX3 LIM-class homeodomain site, PRD=paired-class homeodomain site, BIC=bicoid-class homeodomain site, PIT=Pit-1 POU-class homeodomain site, MD=MyoD basic helix-loop-helix site. The upper panel shows the input Hop protein (^35^S-labeled); the lower panel shows the migration of ^32^P-labeled DNA.

Because amino acids thought to be critical for DNA binding are altered in the *Hop* sequence, Hop protein was tested for its ability to bind DNA by electrophoretic mobility shift assay (EMSA). ^35^S-radiolabeled Hop protein was synthesized by in vitro transcription/translation and then incubated with ^32^P-radiolabeled DNA probes representing LIM-, paired- bicoid-, and POU-class homeodomain binding sites, or a MyoD basic helix-loop-helix protein binding site. In a parallel positive control, the LIM class site was bound by M2-LHX3 [37]. In agreement with other studies on mouse Hop [21,22], interaction between chicken Hop and DNA was not observed (Figure 2). DNA binding also was not observed in similar experiments using bacterially expressed Hop protein (data not shown).

**Figure 3:**
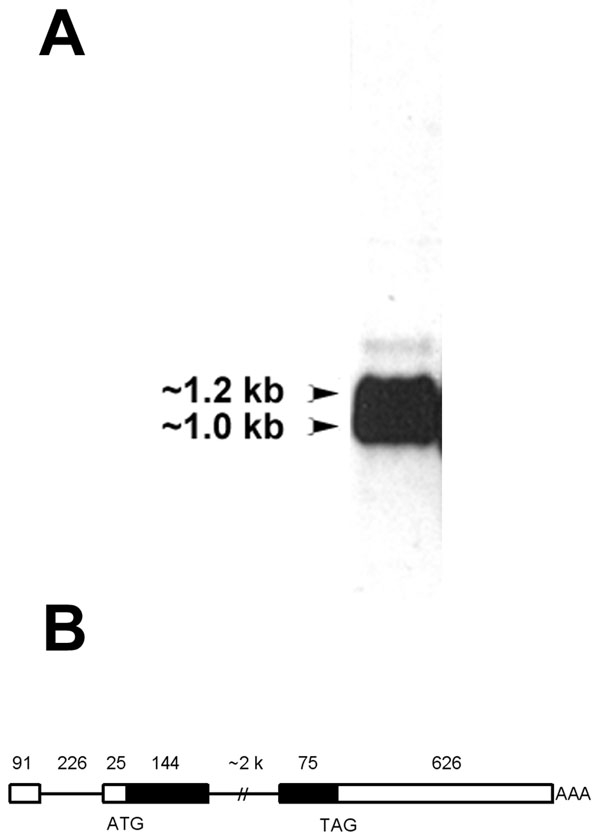
Splicing of *Hop* transcripts in the lens. **A**: Northern blot of total RNA extracted from E15-E16 lens fiber masses and probed with a digoxigenin-labeled *Hop* riboprobe. Two bands were detected that were the approximate predicted size of the *Hop* mRNA, with or without the inclusion of the first intron. **B**: Diagram showing the chicken *Hop* gene structure. The dimensions of the different regions of the gene are not to scale. The numerals above the line diagram mark the number of nucleotide pairs in each region. Introns are represented by thin solid lines and exons by boxes. Filled boxes represent translated regions of the mRNA and unfilled boxes are the untranslated regions.

Sequencing *Hop* PCR products and over 6 kb of the chicken *Hop* genomic locus identified a 226 bp intron in the 5' untranslated region and a second intron of about 2,000 bp located between the regions coding for the first and second alpha helical regions of the Hop homeodomain. This is consistent with our northern blot analysis of lens RNA, which detected two transcripts of about 1 and 1.2 kb (Figure 3A). Sequencing of several *Hop* clones revealed that some *Hop* transcripts lack the first intron, while others may be initiated within the first intron. However, it is possible that these clones represent unspliced transcripts that did not extend to the 5' end of the cDNA. Alternative splicing of the first intron was later confirmed by examination of the chicken genomic sequence using the UCSC genome browser, which shows that some *Hop* ESTs from the chicken genome initiative include the first intron while others do not [38]. This analysis also demonstrates that *Hop* maps to chicken chromosome 4 [38]. The structure of the chicken *Hop* locus is shown in Figure 3B.

**Figure 4:**
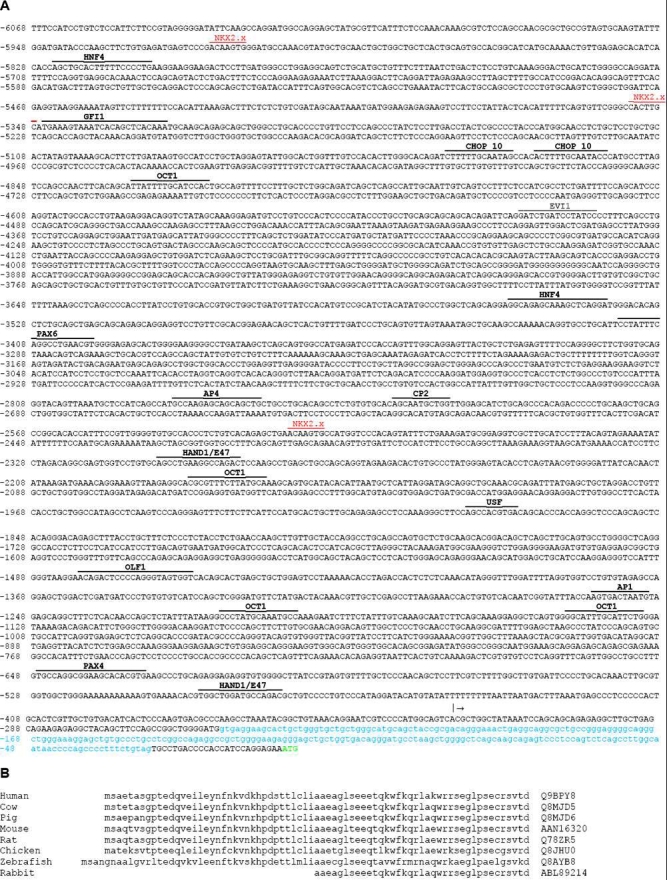
Analysis of the *Hop* gene and protein. The upstream genomic sequence of *Hop* and alignment of the Hop protein sequences from several species. **A**: About 6 kb of DNA sequence upstream of the Hop protein coding sequence, including the first intron (lower case, light blue letters), was annotated with potential transcription factor binding sites, as determined using the P-Match search tool. Settings for the search were adjusted to reveal only the most conservative matches (to minimize false positives). Because Nkx2.x factors regulate Hop expression in the heart and lung, we also show potential Nkx2.x binding sequences (in red), although these motifs were not detected by P-Match when set to minimize false positive matches. The transcription start site of the longest spliced form of Hop mRNA is marked by a vertical bar followed by an arrow. The initial methionine codon is shown in green. **B**: Alignment of the Hop protein sequences from several species. GenBank accession numbers are shown after each sequence. The chicken protein sequence obtained by conceptual translation of the cDNAs sequenced in this study was identical to that in GenBank. The partial rabbit sequence was determined using degenerate PCR primers, since this sequence was not determined previously.

The genomic sequence of *Hop* was analyzed using a search program that identifies putative transcription factor binding sites (P-Match). The sites upstream of the translation start site that were identified in this search are shown in Figure 4A.

To determine whether *Hop* was expressed in the lenses of other species, we used specific or degenerate PCR primers to amplify cDNA prepared from human, mouse, rat, rabbit, and bovine lens fiber cells. The PCR products were sequenced to confirm that *Hop* transcripts were detected in the lenses of each of the species examined. *Hop* cDNA or genomic DNA has not previously been sequenced from rabbits. The sequence of partial *Hop* transcripts from the rabbit lens was submitted to GenBank (accession number EF154428). An alignment of all known Hop protein sequences is shown in Figure 4B.

**Figure 5:**
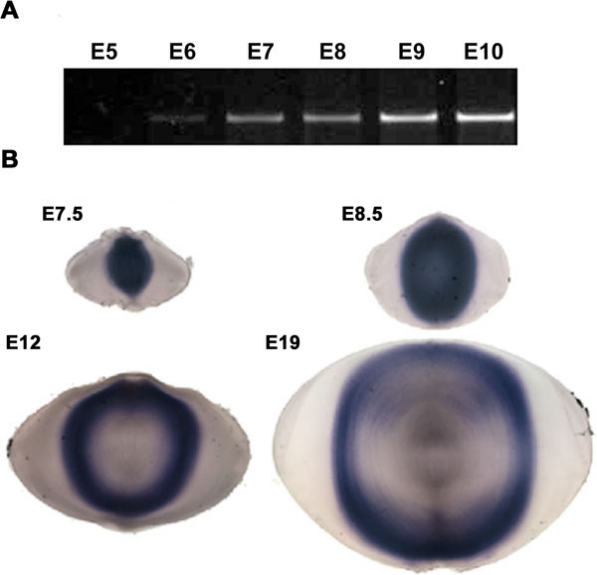
*Hop* expression during lens development. **A**: PCR amplification of *Hop* sequences in RNA extracted from chicken lenses from E5 through E10. *Hop* transcripts were first detectable at E6. Transcript levels increased at later stages. **B**: *Hop* is expressed soon after primary and secondary fiber cells detach from the capsule. In situ hybridization showing the distribution of *Hop* transcripts during lens development in chicken embryos. Sections are from lenses at E7.5, E8.5, E12, and E19. The decreased staining in the center of lenses at E12 and E19 probably reflects a decrease in probe penetration, not a decrease in *Hop* transcripts, because PCR analysis of microdissected lens cores from lenses at these stages revealed no obvious decrease in *Hop* sequences.

The expression and distribution of *Hop* transcripts in the chicken embryo lens were examined using RT-PCR and in situ hybridization. *Hop* sequences were first detected by PCR in cDNA prepared from E6 (Hamburger-Hamilton Stage 28-30) lenses and were readily detected in the fiber cells from older lenses (Figure 5A). A previous study found that primary fiber cells detach from the lens capsule between E5 and E6 [39]. *Hop* mRNA was first detected by situ hybridization at E7.5 in the central fiber cells (Figure 5B). After E7, an increasing number of cells in the central region of the fiber mass expressed *Hop* mRNA. Examination of lens sections suggested that, independent of the age of the lens, *Hop* transcripts were first detected in fiber cells soon after they detached from the lens capsule (Figure 5B).

**Figure 6:**
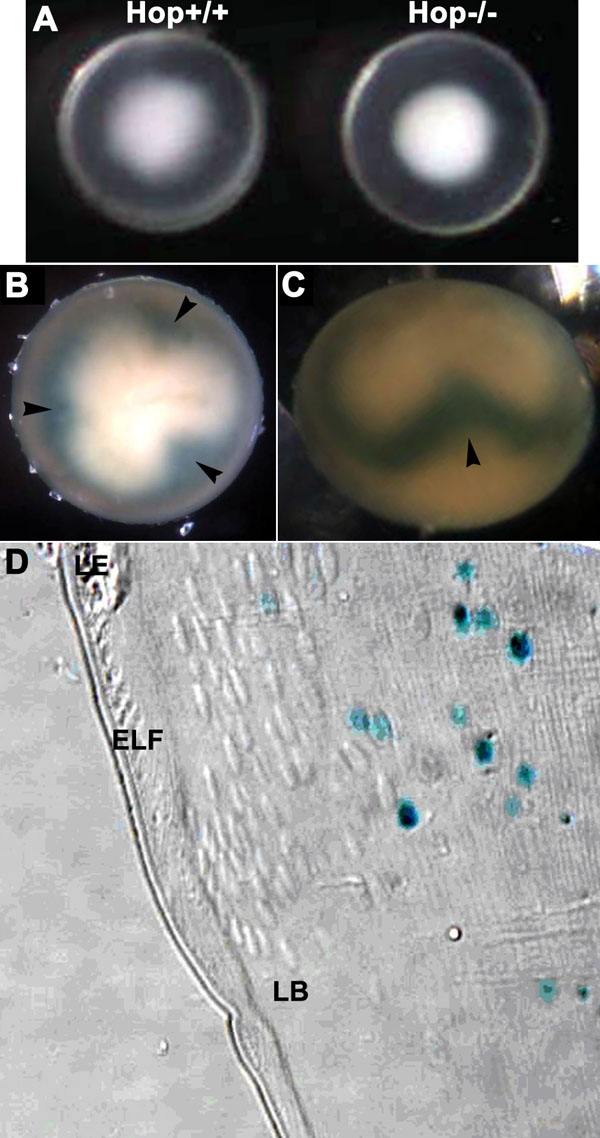
Appearance of *Hop* knockout lenses. *Hop* is expressed in maturing secondary fiber cells in the mouse lens. **A**: *Hop* wild type and null lenses from P3 mice. Both lenses have cold cataracts, as expected of lenses at this age. No consistent variations were detected in the size of the wild type and knockout lenses or in the extent of the cold cataracts. **B**: Polar view of a whole, *Hop* null lens stained for β-galactosidase activity. The superficial zone of the lens has no stained nuclei. The "trefoil" pattern of stained nuclei in the deeper fiber cells is due to the displacement of the nuclei in a more anterior or posterior direction as a result of differences in the extension of the fiber cells toward the anterior and posterior sutures [58]. **C**: The displacement of β-galactosidase-stained nuclei as viewed from the lens equator. **D**: A 1 μm plastic section of the equatorial region of a mouse lens in which both *Hop* alleles were disrupted by the insertion of the sequence encoding nuclear-targeted β-galactosidase [22]. The lens was stained for β-galactosidase activity, embedded in glycol methacrylate, and sectioned. The section was viewed using differential interference contrast optics to show the location of the nuclei of the fiber cells. Only the nuclei of the deeper fiber cells are stained blue, indicating that *Hop* expression is initiated late in fiber cell maturation. β-Galactosidase activity was still present in the fragments of nuclei remaining after organelle loss. The morphology of the cells of the *Hop* knockout lenses appears similar to wild type.

Mouse lenses in which both alleles of *Hop* had been disrupted by insertion of a nuclear-targeted lacZ sequence appeared normal in size (Figure 6A) and were transparent throughout adult life (not shown). When stained for β-galactosidase activity, these lenses revealed a similar pattern of Hop expression as seen in chicken embryo lenses. β-Galactosidase staining was not present in superficial fiber cell nuclei, but was detected in the nuclei of fiber cells that were deep in the fiber mass (Figure 6B-D). β-Galactosidase continued to be present in these nuclei until they were degraded during organelle deletion.

**Figure 7:**
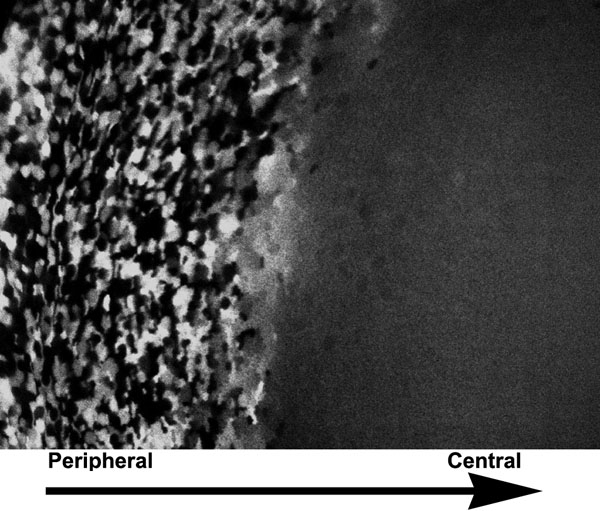
Fiber cell fusion in a *Hop* null lens. During their maturation in *Hop* knockout lenses, fiber cells fuse with their neighbors. This section of a TgN(GFPU)5Nagy; Hop^-/-^ lens is cut perpendicular to the long axis of the fiber cells. GFP fluorescence is seen in a mosaic pattern in the peripheral fiber cells, similar to the pattern described previously for TgN(GFPU)5Nagy lenses that are wild type for *Hop* [24]. Deeper in the fiber mass, GFP fluorescence abruptly spreads to all cells, an indication of fiber cell fusion. This result shows that Hop is not required for the cell-cell fusion of fiber cells during their maturation.

The TgN(GFPU)5Nagy strain of transgenic mice was used to determine whether, during their maturation, fiber cells fused with their neighbors. Mice of this strain express GFP in a mosaic pattern in superficial, elongating fiber cells [24]. When fiber cells fuse during maturation, all cells become uniformly fluorescent, since GFP can now diffuse between neighboring cells. Our results demonstrated that elongating fiber cells of *Hop* knockout lenses showed mosaic expression of GFP, but fiber cells deeper in the lens were uniformly fluorescent (Figure 7). This indicates that the maturing fiber cells of *Hop* null lenses fused with their neighbors during their maturation in a manner that closely resembled that seen in lenses that contained both wild type *Hop* alleles (Figure 7) [24].

## Discussion

We postulated that the proteins that are encoded by transcripts that first appear after lens fiber cells detach from the capsule might be important for fiber cell maturation as well as denucleation. We and others identified transcripts that are differentially accumulated late in fiber cell differentiation [9,20]. It is not known whether these transcripts change in abundance due to increased rates of synthesis or decreased degradation. Vinculin and paxillin mRNAs increase markedly in fiber cells after they detach from the capsule, compared to fiber cells that were still elongating and were attached to the capsule [9]. However, the expression of vinculin and paxillin is not unique to mature fiber cells; these transcripts are expressed at lower levels in elongating fiber cells.

To identify transcripts that are expressed only in fiber cells that have detached from the capsule, we prepared libraries from microdissected lens regions and performed subtractive hybridization. One of the transcripts detected in this screen encoded chicken Hop, an unusual homeodomain-containing protein. Other labs found that *Hop* is prominently expressed in the heart [21,22,34]. Based on its unusual coding sequence, these groups named it "homeodomain only protein" (*Hop*) [21,22] or "odd box" (*OB1*) [34]. *Hop* is the first gene to be identified that is not expressed in elongating fiber cells but is transcribed after fiber cells detach from the lens capsule. This pattern of gene expression demonstrates that there are mechanisms to initiate transcription at this critical stage of lens fiber cell differentiation and raises the possibility that other genes may be similarly regulated.

*Hop* transcripts appear in fiber cells soon after they detach from their basal lamina, the lens capsule. There is ample precedent for the activation of a new gene expression program in other types of epithelial cells after they separate from their basal laminae. For example, when keratinocytes detach from the epidermal basal lamina and move out of the germinative layer of the epidermis, they initiate a complex program of differentiation that is related to the ability of superficial keratinocytes to protect the body surface from desiccation, injury and infection [40]. Thus, *Hop* expression in maturing fiber cells may be regulated by signals from integrins or other matrix-binding proteins [41,42] that are altered following detachment from the capsule.

Previous studies found that Hop positively and negatively modulates gene expression in the heart and lung. In heart muscle [34], Hop reduces transcriptional activation by serum response factor by recruiting histone deacetylases (HDACs) to the promoters of several heart muscle-specific genes [21,22,43]. Hop also functions prominently in the atrium and in the cardiac conduction system, where loss of Hop function results in an abnormal electrocardiogram, associated with a marked and selective reduction in the expression of connexin40 [44]. In the airway epithelium, Hop suppresses surfactant production in type II pneumocytes, again by recruiting HDACs to surfactant protein genes [45]. Hop may function in a similar manner in the lens, perhaps by regulating the expression of crystallin or connexin genes.

In the heart and lung, the expression of *Hop* is regulated by members of the Nkx2.x and GATA families of transcription factors [21,22,45]. Of the several members of these families, none was detectable in whole, adult mouse lens fibers by microarray analysis (Vasiliev, Wang, and Beebe, unpublished). Whether these proteins are expressed at sufficient levels to contribute to Hop expression in the lens remains to be tested.

Analysis of the genomic sequence upstream of the *Hop* coding sequence identified few potential binding sites for transcription factors considered to be key for regulating gene expression during lens fiber cell differentiation (Pax6, c-maf, L-maf, Prox1, Sox1-3, RAR/RXR) [46-49]. There is a potential Pax6 binding element 3.4 kb upstream of the translation start site. However, Pax6 levels decline sharply during fiber cell differentiation [50,51], making it unlikely that Pax6 contributes to the regulation of *Hop* late in fiber cell differentiation. Since *Hop* is the first gene known to be expressed exclusively during the latest phase of fiber cell differentiation, it is not surprising that it may not be regulated in the same manner as genes expressed early in fiber cell formation.

In spite of the paucity of binding sites for these "core" lens fiber cell transcription factors, a CP2 binding site is located at position -2876. CP2 is a ubiquitous factor that was shown to be essential for lens-specific expression of α-crystallin in the chicken [52]. Similarly, USF1, which is expressed in lens cells and regulates the expression of the chicken and mouse αA-crystallin genes [53,54], may regulate *Hop* expression by binding the USF site at -2042. The HAND1/E47 E2 boxes at -559 and -2352 bind basic helix-loop-helix transcription factors and might be negatively regulated by the δ-crystallin enhancer-binding protein, δEF1, which competes for E2 sites [55]. In addition, there are two CHOP10 (C/EBP homologous protein 10) binding sites beginning at position -5184. These are of interest because CHOP10 dimerizes with other members of the C/EBP family of transcription factors to inhibit their activity. C/EBP family members can heterodimerize with ATF4 (CREB2), which is required for the differentiation of secondary lens fiber cells [56]. In a preliminary microarray study, CHOP10 transcripts were decreased tenfold in *Hop* knockout mice, compared to wild type (Vasiliev, Wang, and Beebe, unpublished). This raises the possibility that CHOP10 and Hop are mutual regulators of their respective genes. Since CHOP10 is most often a negative regulator of transcription, it may serve as a feedback regulator of *Hop* expression. The importance of these cis-binding elements in regulating *Hop* expression in the lens and the basis of CHOP10 regulation by Hop will have to be evaluated in future experiments.

Examination of EST databases and staining with specific antibodies showed that, in addition to the cardiac and pulmonary systems, Hop is expressed in many tissues [34,57]. However, other than in the heart and lungs, no defects have been described in *Hop* knockout mice. We observed no obvious phenotype in the lenses of *Hop* null mice. *Hop* null lenses were clear and of normal size and their cellular morphology appeared normal. Fiber cells lacking *Hop* fused with their neighbors and degraded their nuclei in a manner that was morphologically indistinguishable from wild-type lenses. Although *Hop* does not appear to have an essential function in maturing lens fiber cells, it may be possible to use *Hop* regulatory sequences to target the expression of exogenous genes to fiber cells at the stage just before they fuse and degrade their nuclei.

## References

[1] Bloemendal H, de Jong W, Jaenicke R, Lubsen NH, Slingsby C, Tardieu A (2004). Ageing and vision: structure, stability and function of lens crystallins.. Prog Biophys Mol Biol.

[2] Bok D, Dockstader J, Horwitz J (1982). Immunocytochemical localization of the lens main intrinsic polypeptide (MIP26) in communicating junctions.. J Cell Biol.

[3] Sawada K, Agata J, Eguchi G, Quinlan R, Maisel H (1995). The predicted structure of chick lens CP49 and a variant thereof, CP49ins, the first vertebrate cytoplasmic intermediate filament protein with a lamin-like insertion in helix 1B.. Curr Eye Res.

[4] Kato K, Shinohara H, Kurobe N, Goto S, Inaguma Y, Ohshima K (1991). Immunoreactive alpha A crystallin in rat non-lenticular tissues detected with a sensitive immunoassay method.. Biochim Biophys Acta.

[5] Sinha D, Esumi N, Jaworski C, Kozak CA, Pierce E, Wistow G (1998). Cloning and mapping the mouse Crygs gene and non-lens expression of [gamma]S-crystallin.. Mol Vis.

[6] Mulders JW, Voorter CE, Lamers C, de Haard-Hoekman WA, Montecucco C, van de Ven WJ, Bloemendal H, de Jong WW (1988). MP17, a fiber-specific intrinsic membrane protein from mammalian eye lens.. Curr Eye Res.

[7] Wang X, Garcia CM, Shui YB, Beebe DC (2004). Expression and regulation of alpha-, beta-, and gamma-crystallins in mammalian lens epithelial cells.. Invest Ophthalmol Vis Sci.

[8] Bassnett S, Winzenburger PA (2003). Morphometric analysis of fibre cell growth in the developing chicken lens.. Exp Eye Res.

[9] Beebe DC, Vasiliev O, Guo J, Shui YB, Bassnett S (2001). Changes in adhesion complexes define stages in the differentiation of lens fiber cells.. Invest Ophthalmol Vis Sci.

[10] Shestopalov VI, Bassnett S (2000). Expression of autofluorescent proteins reveals a novel protein permeable pathway between cells in the lens core.. J Cell Sci.

[11] Bassnett S, Beebe DC (1992). Coincident loss of mitochondria and nuclei during lens fiber cell differentiation.. Dev Dyn.

[12] Kuwabara T, Imaizumi M (1974). Denucleation process of the lens.. Invest Ophthalmol.

[13] Vrensen GF, Graw J, De Wolf A (1991). Nuclear breakdown during terminal differentiation of primary lens fibres in mice: a transmission electron microscopic study.. Exp Eye Res.

[14] Bassnett S (1995). The fate of the Golgi apparatus and the endoplasmic reticulum during lens fiber cell differentiation.. Invest Ophthalmol Vis Sci.

[15] Bassnett S (1997). Fiber cell denucleation in the primate lens.. Invest Ophthalmol Vis Sci.

[16] Bassnett S, Mataic D (1997). Chromatin degradation in differentiating fiber cells of the eye lens.. J Cell Biol.

[17] Bassnett S (2002). Lens organelle degradation.. Exp Eye Res.

[18] Overbeek PA, Chepelinsky AB, Khillan JS, Piatigorsky J, Westphal H (1985). Lens-specific expression and developmental regulation of the bacterial chloramphenicol acetyltransferase gene driven by the murine alpha A-crystallin promoter in transgenic mice.. Proc Natl Acad Sci USA.

[19] Duncan MK, Li X, Ogino H, Yasuda K, Piatigorsky J (1996). Developmental regulation of the chicken beta B1-crystallin promoter in transgenic mice.. Mech Dev.

[20] Ivanov D, Dvoriantchikova G, Pestova A, Nathanson L, Shestopalov VI (2005). Microarray analysis of fiber cell maturation in the lens.. FEBS Lett.

[21] Chen F, Kook H, Milewski R, Gitler AD, Lu MM, Li J, Nazarian R, Schnepp R, Jen K, Biben C, Runke G, Mackay JP, Novotny J, Schwartz RJ, Harvey RP, Mullins MC, Epstein JA (2002). Hop is an unusual homeobox gene that modulates cardiac development.. Cell.

[22] Shin CH, Liu ZP, Passier R, Zhang CL, Wang DZ, Harris TM, Yamagishi H, Richardson JA, Childs G, Olson EN (2002). Modulation of cardiac growth and development by HOP, an unusual homeodomain protein.. Cell.

[23] Huang JX, Feldmeier M, Shui YB, Beebe DC (2003). Evaluation of fibroblast growth factor signaling during lens fiber cell differentiation.. Invest Ophthalmol Vis Sci.

[24] Shestopalov VI, Bassnett S (2003). Development of a macromolecular diffusion pathway in the lens.. J Cell Sci.

[25] Vasiliev OL, Lukyanov SA, Belyavsky AV, Kazanskaya OV, Zaraisky AG (1997). A novel marker of early epidermal differentiation: cDNA subtractive cloning starting on a single explant of Xenopus laevis gastrula epidermis.. Int J Dev Biol.

[26] Zaraisky AG, Lukyanov SA, Vasiliev OL, Smirnov YV, Belyavsky AV, Kazanskaya OV (1992). A novel homeobox gene expressed in the anterior neural plate of the Xenopus embryo.. Dev Biol.

[27] Chomczynski P, Sacchi N (1987). Single-step method of RNA isolation by acid guanidinium thiocyanate-phenol-chloroform extraction.. Anal Biochem.

[28] Barnes WM (1994). PCR amplification of up to 35-kb DNA with high fidelity and high yield from lambda bacteriophage templates.. Proc Natl Acad Sci USA.

[29] Bridwell JA, Price JR, Parker GE, McCutchan Schiller A, Sloop KW, Rhodes SJ (2001). Role of the LIM domains in DNA recognition by the Lhx3 neuroendocrine transcription factor.. Gene.

[30] Wilson D, Sheng G, Lecuit T, Dostatni N, Desplan C (1993). Cooperative dimerization of paired class homeo domains on DNA.. Genes Dev.

[31] Saadi I, Semina EV, Amendt BA, Harris DJ, Murphy KP, Murray JC, Russo AF (2001). Identification of a dominant negative homeodomain mutation in Rieger syndrome.. J Biol Chem.

[32] Rhodes SJ, Chen R, DiMattia GE, Scully KM, Kalla KA, Lin SC, Yu VC, Rosenfeld MG (1993). A tissue-specific enhancer confers Pit-1-dependent morphogen inducibility and autoregulation on the pit-1 gene.. Genes Dev.

[33] Harland R. In situ hybridization: an improved whole-mount method for Xenopus embryos. In: Kay BK, Peng HB, editors. Methods in Cell Biology. Vol. 36. San Diego: Academic Press; 1991. p. 685-695. 10.1016/s0091-679x(08)60307-6 1811161

[34] Adu J, Leong FT, Smith NR, Leek JP, Markham AF, Robinson PA, Mighell AJ (2002). Expression of mOb1, a novel atypical 73 amino acid K50-homeodomain protein, during mouse development.. Gene Expr Patterns.

[35] Burglin TR. A Comprehensive Classification of Homeobox Genes. In: Duboule D, editor. Guidebook to the Homeobox Genes. New York: Oxford University Press; 1994. p. 25-71.

[36] Yasui K, Zhang S, Uemura M, Saiga H (2000). Left-right asymmetric expression of BbPtx, a Ptx-related gene, in a lancelet species and the developmental left-sidedness in deuterostomes.. Development.

[37] Sloop KW, Dwyer CJ, Rhodes SJ (2001). An isoform-specific inhibitory domain regulates the LHX3 LIM homeodomain factor holoprotein and the production of a functional alternate translation form.. J Biol Chem.

[38] International Chicken Genome Sequencing Consortium Sequence and comparative analysis of the chicken genome provide unique perspectives on vertebrate evolution. Nature 2004 432 695 716Erratum inNature 2005 433 777 15592404 10.1038/nature03154

[39] Shestopalov VI, Bassnett S (2000). Three-dimensional organization of primary lens fiber cells.. Invest Ophthalmol Vis Sci.

[40] Presland RB, Dale BA (2000). Epithelial structural proteins of the skin and oral cavity: function in health and disease.. Crit Rev Oral Biol Med.

[41] Menko S, Philp N, Veneziale B, Walker J (1998). Integrins and development: how might these receptors regulate differentiation of the lens.. Ann N Y Acad Sci.

[42] Duncan MK, Kozmik Z, Cveklova K, Piatigorsky J, Cvekl A (2000). Overexpression of PAX6(5a) in lens fiber cells results in cataract and upregulation of (alpha)5(beta)1 integrin expression.. J Cell Sci.

[43] Kook H, Lepore JJ, Gitler AD, Lu MM, Wing-Man Yung W, Mackay J, Zhou R, Ferrari V, Gruber P, Epstein JA (2003). Cardiac hypertrophy and histone deacetylase-dependent transcriptional repression mediated by the atypical homeodomain protein Hop.. J Clin Invest.

[44] Ismat FA, Zhang M, Kook H, Huang B, Zhou R, Ferrari VA, Epstein JA, Patel VV (2005). Homeobox protein Hop functions in the adult cardiac conduction system.. Circ Res.

[45] Yin Z, Gonzales L, Kolla V, Rath N, Zhang Y, Lu MM, Kimura S, Ballard PL, Beers MF, Epstein JA, Morrisey EE (2006). Hop functions downstream of Nkx2.1 and GATA6 to mediate HDAC-dependent negative regulation of pulmonary gene expression.. Am J Physiol Lung Cell Mol Physiol.

[46] Lang RA (2004). Pathways regulating lens induction in the mouse.. Int J Dev Biol.

[47] Gopal-Srivastava R, Cvekl A, Piatigorsky J (1998). Involvement of retinoic acid/retinoid receptors in the regulation of murine alphaB-crystallin/small heat shock protein gene expression in the lens.. J Biol Chem.

[48] Yoshida T, Yasuda K (2002). Characterization of the chicken L-Maf, MafB and c-Maf in crystallin gene regulation and lens differentiation.. Genes Cells.

[49] Wigle JT, Chowdhury K, Gruss P, Oliver G (1999). Prox1 function is crucial for mouse lens-fibre elongation.. Nat Genet.

[50] Duncan MK, Xie L, David LL, Robinson ML, Taube JR, Cui W, Reneker LW (2004). Ectopic Pax6 expression disturbs lens fiber cell differentiation.. Invest Ophthalmol Vis Sci.

[51] Duncan MK, Haynes JI, Cvekl A, Piatigorsky J (1998). Dual roles for Pax-6: a transcriptional repressor of lens fiber cell-specific beta-crystallin genes.. Mol Cell Biol.

[52] Murata T, Nitta M, Yasuda K (1998). Transcription factor CP2 is essential for lens-specific expression of the chicken alphaA-crystallin gene.. Genes Cells.

[53] Cvekl A, Sax CM, Bresnick EH, Piatigorsky J (1994). A complex array of positive and negative elements regulates the chicken alpha A-crystallin gene: involvement of Pax-6, USF, CREB and/or CREM, and AP-1 proteins.. Mol Cell Biol.

[54] Sax CM, Cvekl A, Piatigorsky J (1997). Transcriptional regulation of the mouse alpha A-crystallin gene: binding of USF to the -7/+5 region.. Gene.

[55] Sekido R, Murai K, Funahashi J, Kamachi Y, Fujisawa-Sehara A, Nabeshima Y, Kondoh H (1994). The delta-crystallin enhancer-binding protein delta EF1 is a repressor of E2-box-mediated gene activation.. Mol Cell Biol.

[56] Tanaka T, Tsujimura T, Takeda K, Sugihara A, Maekawa A, Terada N, Yoshida N, Akira S (1998). Targeted disruption of ATF4 discloses its essential role in the formation of eye lens fibres.. Genes Cells.

[57] Muhlfriedel S, Kirsch F, Gruss P, Stoykova A, Chowdhury K (2005). A roof plate-dependent enhancer controls the expression of Homeodomain only protein in the developing cerebral cortex.. Dev Biol.

[58] Kuszak JR, Zoltoski RK, Tiedemann CE (2004). Development of lens sutures.. Int J Dev Biol.

